# Does the Venus de Milo have a Spinal Deformity?

**DOI:** 10.7759/cureus.3219

**Published:** 2018-08-28

**Authors:** Kevlian Andrew, Joe Iwanaga, Marios Loukas, Jens Chapman, Rod J Oskouian, R. Shane Tubbs

**Affiliations:** 1 Anatomical Sciences, St. George's University, St. George, GRD; 2 Medical Education and Simulation, Seattle Science Foundation, Seattle, USA; 3 Anatomical Sciences, St. George's University, St. George's, GRD; 4 Orthopedics Spine Surgery, Swedish Neuroscience Institute, Seattle, USA; 5 Neurosurgery, Swedish Neuroscience Institute, Seattle, USA; 6 Neurosurgery, Seattle Science Foundation, Seattle, USA

**Keywords:** venus de milo, aphrodite, disability, asymmetry

## Abstract

The Venus de Milo, an ancient Greek statue, has been viewed as one of the most celebrated pieces of art in Western culture. It was sculpted during the Hellenistic period between 150 and 50 BC and is believed to be the work of Alexandros of Antioch. The sculpture is thought to depict Aphrodite, the Greek goddess of love and beauty. When assembled, the two halves of the sculpture meet in an almost horizontal line that is purposefully obscured by a roll of garment around the hips. It has been noted that the midline of the statue’s face is displaced slightly. German anatomist von Henke observed that Venus’s pelvis is obliquely positioned and that there is a leg length discrepancy. These findings lead him and others to posit that the Venus de Milo might have a subtle spinal deformity. In this review, we examine the literature regarding this famous statue and evidence that the model of the statue might have had a deformity of the vertebral column.

## Introduction and background

**Figure 1:**
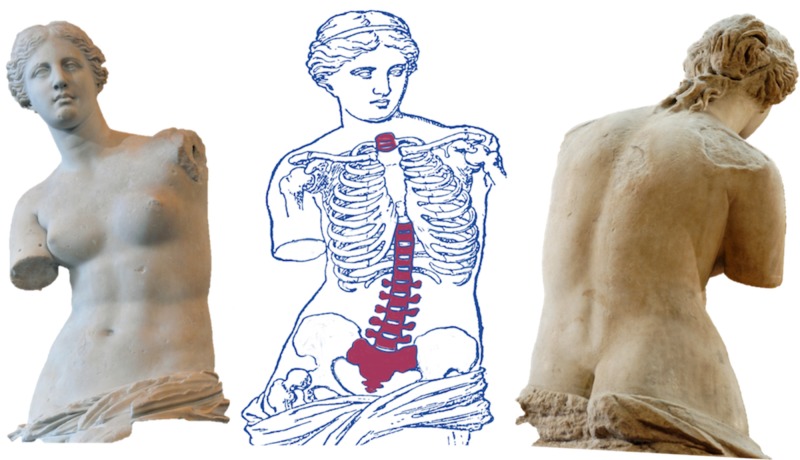
Left: anterior view of the Venus de Milo; Middle: schematic drawing illustrating the possible abnormal curvature of the spine; Right: posterior view of the Venus de Milo

The Venus de Milo, an ancient Greek marble statue, has been viewed as one of the most celebrated pieces of art in Western culture [1-2] (Figure 1). The statue was sculpted during the Hellenistic period between 150 and 50 BC. The Venus de Milo has been considered an ageless ideal of feminine beauty [3]. It is a depiction of Aphrodite [4], the Greek goddess of sexual love, fertility, and beauty [4-5]. Aphrodite in Greek culture is identified as Venus by the Romans hence, the statue is often referred to as Aphrodite de Milos [5]. The goddess Venus was thought to rule over love and beauty but was notoriously known to be promiscuous and vain [2]. The statue has been thought to represent the triumph of the goddess in the Judgement of Paris [6-7]. It is thought to most likely have been housed in an alcove in a civic gymnasium [1,6-7].

Although seen as the universal symbol of artistic beauty [4], the statue has been found to have asymmetries suggesting to some that the model may have had some form of spinal deformity such as scoliosis. This article aims to explore such opinions on the sculpture and its history, to identify whether or not the original model possessed features suggestive of scoliosis or other deformities of the spine.

## Review

Discovery of the statue

The statue was discovered in 1820 on the Greek island of Melos. Given that the Greek word for apple is ‘melos,’ and the island is said to have had a natural outline resembling that of an apple [6], it is thought that the name of the statue alludes to its home on the island [7]. The statue was discovered by a farmer who was removing stones from an ancient wall that previously encircled a gymnasium [6]. Upon digging, two main pieces of carved marble were found [1]. The upper torso was discovered without arms [2,6] and the left foot was also noted to be missing from the lower portion [6]. These were later put together to form an armless female statue [1]. Fragments discovered along with the main pieces included two stone pillars [1,7] and a left hand holding an apple. Soon after the excavation, the sculpture was recognized as a monumental find [8].

However, several other imperfections were found on the statue [6]. These included scratches on its brow, nose, chin, and missing earlobes [6]. There was evidence of prior restoration on the hip to replace larger pieces that had broken off [6].

Further digging revealed a base, and numerous loose marble fragments [6], including small pieces of drapery and hair [7] that had also broken off. The Venus’s left hand was discovered with the fragments but was never attached to the body because it was less finished than other parts [9]. It is reported that some of the fragments recovered with the statue were shipped to France but went missing soon after arrival at the Louvre. Among these was thought to be the left hand holding an apple [2], an upper arm [7] and a piece of the plinth (base of the sculpture), where the sculptor Agesandros (Alexandros) of Antioch had carved his name [2,7]. Shortly after discovery, the statue was bought from Mahmud II, the Ottoman Emperor [6], for 550 francs [6] (about $50) by the French ambassador Marquis de Rivie`re [1,6]. The statue was given as a gift to Louis XVIII, king of France in 1820. The king donated the statue to the Louvre Museum in Paris where it is still on display today [1-4,6-8]. Since its arrival to France, the statue has been the absolute emblem of classical beauty [2].

Anatomy of the statue and potential spinal deformity

The larger than life-sized [7] statue stands 6’7” tall. When assembled, the two halves of the sculpture met in an almost horizontal line, purposefully covered by a roll of garment around the hips [6]. The statue’s head and upper body [2,7] are turned slightly toward the left [2,7]. The Venus appears to be slightly bent over while lifting her thigh, as though attempting to prevent her garment from sliding down her leg [2]. Some research suggests that the right hand held her clothes while the left arm rested on a pillar [2]. Others hypothesize that the right arm extended inferiorly across the stomach toward the left, and that a hole beneath the right breast may have once held a tenon to support the weight of the right arm [6]. According to Lethaby, the Venus de Milo probably leaned her arm onto a pillar when first created [10].

An analysis of the Venus de Milo by Goeler von Ravensburg found that the piece represented such outstanding naturalistic art that it was most likely created with the aid of a living model. It was also noted that the midline of the statue’s face was displaced slightly and although other Hellenistic statues displayed subtle asymmetries, the Venus’s were more profound. In 1886, the German anatomist Philipp Jakob Wilhelm von Henke (1834-1896) from the University of Tübingen observed that the pelvis of the Venus was oblique, and that her legs were different lengths. These observations suggested that the model for the statue limped in life and possibly had a spinal deformity [11]. Henke also reported that the two lines connecting the pupils and both lateral ends of the lips were neither perpendicular nor parallel to the nose [3]. Interestingly, Henke is also remembered eponymously for Henke’s space or the retropharyngeal space.

Christoph Hasse, von Henke’s colleague and a fellow at the anatomical institute at the University of Breslau, also performed a study in 1886 where he investigated the face of the Venus de Milo and compared to controls. The most important asymmetries found were that the left eye was closer to the midline than the right and the pupils were not on the same horizontal line. However, these asymmetries were common in controls so a conclusion for a head tilt indicative of a craniospinal deformity could not be made [3]. Hasse also made close observations on the muscles of the spine and shoulder but again, definitive conclusions in regard to spinal deformity could not be made.

Hasse hypothesized that the asymmetry of the pupil line compensates for the frequently observed pelvic asymmetry and slight bowing of the spine, which tilts most individual’s heads to one side of the body. Later, in 1888, Hasse performed a study on the female pelvis. This study found the same asymmetrical pattern of the hips in the studied women as was displayed in the Venus. In 1893, Hasse performed another study on 5,141 men, where asymmetry of the legs and spine were analysed. They found that 16% of men had slight left curvature of the spine, 52% had some right bowing, and only 32% had a completely straight spine. These results supported these authors’ earlier speculation that the non-horizontal eyeline compensated for the tilting of the spine. Thus, they contested the previously held assumption that the human body was externally symmetrical [3].

Inner beauty with anatomical disability

The Venus has been viewed as “an icon of silent feminine beauty” itself becoming synonymous with feminine beauty [1], the ultimate symbol of charm [2]. Despite her fragmentary state on discovery, the Venus was conceived as beautiful and complete. She represents a shift in culture where completeness was considered essential for art to the so-called disability aesthetics where disabled, dismembered, deformed, or diseased likenesses were embraced.

Declared to be the eternal standard of female beauty despite missing parts of her upper limbs, the Venus de Milo is thought by some to be beautified by her flaws [9]. In fact, Louis XVIII accepted recommendations from Quatreme`re de Quincy, a well-known art critic, who had strong feelings against restoring the arms to the statue [4].

## Conclusions

The Venus de Milo, one of the most monumental statues of Western culture, has over time expanded to become associated with disability and inner beauty. Although there is some evidence for a leg length discrepancy and facial asymmetry, most studies aimed at the anatomy of this stature have concluded that the model for the stature is normal with no significant anatomical variations or spinal pathology such as scoliosis.
